# Clinical and radiologic features of extraskeletal myxoid chondrosarcoma including initial presentation, local recurrence, and metastases

**DOI:** 10.2478/raon-2014-0005

**Published:** 2014-07-10

**Authors:** Neena Kapoor, Atul B. Shinagare, Jyothi P. Jagannathan, Shaan H. Shah, Katherine M. Krajewski, Jason L. Hornick, Nikhil H. Ramaiya

**Affiliations:** Department of Imaging, Dana-Farber Cancer Institute/ Brigham and Women’s Hospital, Harvard Medical School, Boston, MA 02115, USA

**Keywords:** extraskeletal myxoid chondrosarcoma, CT, MRI, FDG-PET/CT

## Abstract

**Background:**

The aim of the study was to evaluate the clinical and imaging features of extraskeletal myxoid chondrosarcoma (EMC) including initial presentation, recurrence, and metastases.

**Patients and methods.:**

In this institutional review board-approved retrospective study, imaging features of 13 patients with pathologically proven EMC seen from August 1995 to December 2011 were analyzed. The group included 3 women and 10 men and the mean age was 54 years (range 29–73 years). Imaging studies were evaluated by two radiologists in consensus. Location, size, and imaging features of primary tumors were recorded as well as the presence of recurrent disease and location of metastases.

**Results:**

Among 13 patients, 3 died during the timeframe of this study. Nine patients had primary tumor in the lower extremity, and average tumor size was 9.3 cm (range 3.3–18 cm). On MRI, primary tumors were hyperintense on T2, isointense to muscle on T1, and demonstrated peripheral/septal enhancement. Three patients had local recurrence and 12 had metastatic disease, with lung involvement being the most common. Tumor density on contrast enhanced CT ranged from 8.2 to 82.9 Hounsfield unit (HU). FDG-PET/CT imaging was performed in 3 patients. One patient had no FDG avid disease and 2 patients had metastatic disease with standard uptake values (SUV) of 2.8 and 7.4. The patient with intense FDG uptake demonstrated more solid appearing tumor burden and had the shortest survival.

**Conclusions:**

EMC is a rare tumor that often occurs in the lower extremities and frequently metastasizes to the lungs. Increased tumor density and increased FDG uptake may be related to more aggressive disease.

## Introduction

Extraskeletal myxoid chondrosarcoma (EMC) is a rare soft tissue tumor characterized by uniform spindle cells arranged in a reticular growth pattern in abundant myxoid stoma.^1^,^2^ Considered to be slow growing, it generally arises in the deep soft tissues of the proximal limbs but several unusual sites such as the scrotum and finger have been documented.^3^–^5^ The typical appearance is of a lobulated mass composed of gelatinous nodules with internal fibrous septa.^6^ Although originally believed to be a variant of chondrosarcoma, the World Health organization has classified it as a tumor of uncertain differentiation due to its lack of cartilaginous differentiation.^3^ Additionally, cytogenetic studies have shown that EMC is a unique entity with a reciprocal translocation, t(9;22) (q22;q12), not seen in conventional skeletal chondrosarcoma.^7^–^12^ Despite being considered a low grade sarcoma with a prolonged clinical course, extraskeletal myxoid chondrosarcoma has been shown to have a high rate of local recurrence and metastasis.^13^

The literature documenting the imaging features of extraskeletal myxoid chondrosarcoma is limited. With rare exception, most of the radiology literature has consisted of case reports or has been based on a single imaging modality with little evaluation of the imaging spectrum.^3^–^6^ The purpose of this study was to evaluate the imaging features of primary extraskeletal myxoid chondrosarcoma, as well as the imaging characteristics of local recurrence and metastatic disease.

## Patients and methods

### Patients

In this institutional review board-approved retrospective study, the electronic medical records of 13 patients with pathologically proven extraskeletal myxoid chondrosarcoma who were evaluated at our institution from August 1995 to December 2011 were reviewed. No patients with pathologically proved EMC were excluded from the study.

### Image analysis

A systematic review of all imaging studies, including baseline and follow-up studies, was performed by two radiologists with 8 and 13 years of experience in consensus. A total of 7 MRIs, 26 CTs, and 3 FDG-PET/CT studies were analyzed. Four of the 13 patients had MR imaging of their primary tumor at our institution. For these patients the location and size (largest dimension in two orthogonal planes) was documented. The T1, T2, and enhancement characteristics were noted. T2 images with and without fat saturation and STIR images were included when evaluating T2 characteristics of the tumors. The same features were also evaluated in the 3 patients with histologically proven local recurrence.

Twelve patients had metastatic disease, with all undergoing contrast enhanced CT. Two radiologists reviewed the sites and imaging features of metastases in consensus. The Hounsfield unit (HU) of the center of each primary, recurrent, and metastatic site was measured to determine if CT attenuation correlated with the classic pathologic description of abundant myxoid stroma. Density measurements were performed on lung metastases only if tumor opacity on mediastinal window images was greater than half the size of that seen on lung window images. This technique has been used in other studies to correlate CT attenuation of lung nodules with prognosis.^14^,^15^ FDG-PET/CT imaging was performed on three patients with metastatic disease who demonstrated progressive disease on diagnostic restaging CT scans and were enrolled in experimental clinical trials. On FDG-PET/CT, the FDG avidity (standard uptake value [SUV]_max_) of the largest tumor was recorded.

### Histopathologic and clinical correlation

The histology was reviewed by a single pathologist from our institution with expertise in sarcoma. The following pathologic features were recorded: mitotic rate, necrosis, and tumor margin. It is impractical and unnecessary in clinical practice to histologically confirm each metastatic site. However, at least one metastatic site in each of these patients was confirmed by biopsy. The remaining sites of disease were presumed to be metastatic if they showed unequivocal progression on imaging or if they showed treatment response consistent with the overall clinical picture. Other clinical features including primary presentation, treatment offered, recurrence or metastasis-free interval and outcome were also noted.

### Statistical analysis

In order to study the effect of size of the primary tumor on behavior of EMC, we correlated the tumor size (largest dimension in two orthogonal planes) with metastasis-free interval using Spearman correlation. Since in our experience, the majority of EMCs occurred in the extremities, the extremity EMCs were compared with EMC in the torso for differences in size and metastasis-free interval (Mann-Whitney test). Non-parametric tests were used in order to minimize the effect of a few outlying values. We originally intended to analyze the effect of size and location on recurrence-free interval and survival; however, given the small number of patients with local recurrence and patients deceased, we did not perform that analysis.

## Results

### Patients

The patient population consisted of 3 women and 10 men, with a mean age of 54 years (range 29–73 years) (Table 1). Nine patients had their primary tumor in the lower extremity. The site of primary tumor was in the pelvis for 2 patients and in the spine for 2 patients (Table 2). The average follow up interval was 40.5 months (range 7–194 months). One patient was lost to follow up. Three patients had locally recurrent disease and 12 patients had metastases. Three patients died during the time-frame of the study, 1 was lost to follow up, and 9 are still alive.

### Imaging features of primary disease

The average tumor size was 9.3 cm (range 3.3–18 cm). All tumors were large and lobular, with no internal calcification. Four patients had MRI of the primary tumor. Tumors were isointense to muscle on T1, hyperintense to muscle on T2, and contained T2 hypointense internal septa. Primary tumors also demonstrated peripheral/septal enhancement (Figure 1). Contrast enhanced CT imaging was available for 2 primary tumors which were slightly hypodense to muscle with HU measuring 23.4 and 30.2 (Table 3).

### Local recurrence

Three patients had locally recurrent disease. The average time to recurrence was 52.3 months (range 4–81 months). Surveillance imaging varied among patients but generally consisted of CT imaging every 3–6 months. Of the three patients with local recurrence, one patient had routine MRI surveillance every year while the other two patients had MRI based on their clinical situation. One patient had clinical symptoms that lead to an MRI while the other patient has local recurrence that was detected on surveillance CT. Of note, MRI in this case revealed more extensive tumor burden which was underestimated on CT. In all cases, there was no statistically significant correlation between the size of the original tumor and recurrent disease.

The appearance of recurrent disease was similar to that of primary disease. Masses were lobular, extremely T2 hyperintense, isointense on T1, and usually demonstrated T2 hypointense internal septa and peripheral/septal enhancement. The three patients also had contrast enhanced CT imaging of their local recurrence. Two patients had recurrent tumor that was isodense to muscle. The HU of the other patient’s recurrent tumor was 72.4. However, this tumor was immediately adjacent to a femoral prosthesis and measurement was compromised by substantial streak artifact. The density of this tumor was likely closer to that of the patient’s meta-static disease which measured 32.4 to 39.7 HU.

### Metastatic disease

Twelve patients developed metastases. The average time between initial presentation and development of metastatic disease was 7 months (range 0–93 months). Imaging follow up for patients varied but generally consisted of CT staging every 3 to 6 months. There was no correlation between size of the primary tumor and metastasis free interval. All twelve patients had lung metastases, making it the most frequent site of tumor metastasis regardless of original tumor site (Table 2). Bone was the next most common site, though far less likely with only 3 patients developing osseous metastases. Osseous metastases were lytic in all three, and in 2 of the 3 patients were associated with a soft tissue component. Abdominal or pelvic viscera, such as liver, spleen, pancreas, and kidneys, were not involved. Metastatic lesions demonstrated HU ranging from 8.2 to 82.9, with median of 31.2 HU. No correlation was seen between tumor size and metastasis-free interval. No significant difference was seen in tumor size or metastasis-free interval between EMC in the extremity or torso.

**FIGURE 2.**
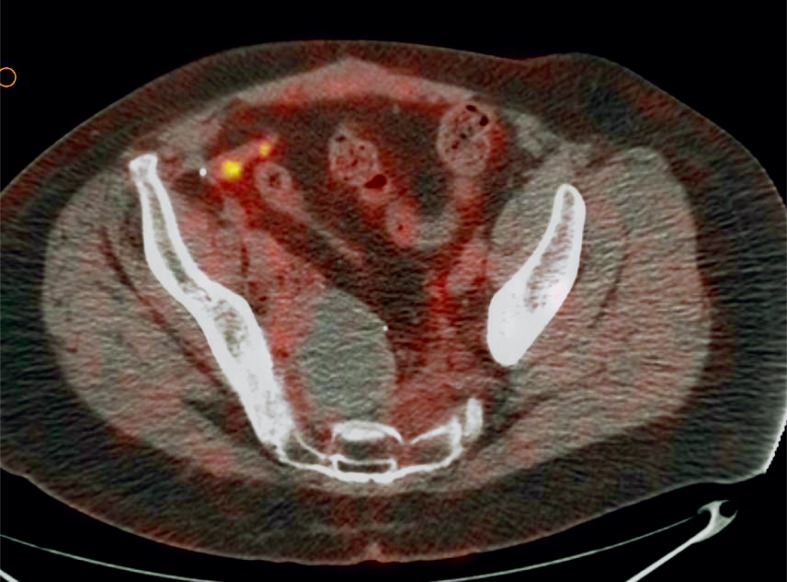
54 year old male with extraskeletal myxoid chondrosarcoma. FDG-PET/CT imaging shows mild peripheral FDG uptake with an standard uptake value (SUV)_max_ of 2.8 in a patient with mildly dense tumor burden.

FDG-PET/CT imaging was performed on three patients with metastases who demonstrated progressive disease on diagnostic restaging CT scans and were enrolled in experimental clinical trials. One patient had non-FDG avid disease and another patient had mild peripheral tumor uptake with an SUV_max_ 2.8 (Figure 1 and 2). The third patient had intense FDG avid disease with an SUV_max_ of 7.4 (Figure 3). During the time period of this study, 2 of these 3 patients died. The patient with intense FDG uptake was also the patient with solid appearing metastatic disease (82.9 HU). This patient died within 18 months of diagnosis. The patient with no significant FDG uptake had low density metastases on contrast enhanced CT (33.6 HU), which is more typical of myxoid tumors, and died 81 months after diagnosis. The patient with mildly FDG avid disease had metastatic tumor measuring 41.0 HU. This patient is still a live, approximately 43 months after diagnosis. Table 4 summarizes the SUV_max_, CT density, and survival after diagnosis for the three patients who obtained FDG-PET/CT imaging.

### Histopathologic correlation

Eleven of the thirteen patients had wide local excision as part of their treatment. The two patients who were not treated surgically had pulmonary metastases at the time of presentation. Three tumors demonstrated necrosis. Mitotic rate was generally low, ranging from 1–4 per 10 high-power fields. Of note, the patient with intense FDG uptake showed high grade tumor. Histologically, tumor cells were arranged in a reticular architecture with abundant myxoid stroma. Tumors were composed of bland and uniform spindle cells with hyperchromatic nuclei and delicate eosinophilic cytoplasm. Fluorescence in situ hybridization (FISH) using break-apart probes showed EWSR1 rearrangement at 22q12 in one case (Figure 4).

### Clinical correlation

Three patients died during the course of this study. All but 2 of the 13 patients were initially treated with surgical resection. The 2 patients who did not receive surgery had pulmonary metastases at the time of presentation. Five patients received chemo-therapy and radiation as additional treatment at some point during their illness. Two received only additional radiation therapy after surgery and 1 received only additional chemotherapy. As mentioned, 1 patient demonstrated particularly aggressive tumor. This patient had a wide local resection of his primary tumor and later went on to receive radiation and chemotherapy.

## Discussion

Extraskeletal myxoid chondrosarcoma is a rare neoplasm. The radiology literature about this entity is sparse, and to our knowledge this is one of the first studies to report the entire imaging spectrum of primary, recurrent, and metastatic disease of patients from a single institution.

Extraskeletal myxoid chondrosarcoma predominantly occurs in the soft tissues of the lower extremities and has a prolonged clinical course.^13^ The mean age of the cohort was 54 years (range 29–73 years), similar to other studies.^2^,^13^ Approximately 54% of patients in this cohort had primary disease in the thigh, which is consistent with prior reported studies. The average tumor size was 9.3 cm with a range of 3.3 to 18 cm (Table 1).

On MRI, all primary tumors were hyperintense on T2, isointense to muscle on T1, and demonstrated peripheral/septal enhancement. These results differ from another study in which the T1 characteristics were predominantly intermediate or high relative to muscle.^6^ This could reflect some degree of variability in the T1 appearance of the tumor.

High T2 signal and heterogeneous enhancement are similar findings to previously reported MRI characteristics of primary tumor.^6^ On contrast enhanced CT, primary tumors were isodense to slightly hypodense to muscle with no internal calcification. Local recurrence occurred in three patients with similar imaging features to the primary tumor. Given that EMC can have a similar density to muscle on CT while appearing extremely hyperintense on T2 weighted MRI, MRI seems to be the best modality for detecting primary disease and local recurrence.

Twelve patients had metastatic disease, with all 12 having at least lung metastases. Bone and nodal involvement was present in several patients. Interestingly, abdominal visceral involvement was not seen. These findings are similar to a prior study which showed that lung metastases were the most common site of metastatic disease.^2^ The total number of patients in our study with metastases was higher than a previous study which reported 13 out of 42 patients either presenting with or developing metastases during an average follow up period of 7.4 years.^13^ The higher level of metastases in our cohort could be due to the fact that our institution is a tertiary care hospital that is a referral center for complicated cases.

Three out of 13 patients died in this study population. This corresponds to the low grade nature of EMC and previous reports of an overall survival of 100% at 5 years and 88% at 10 years.^13^ One of the patients who died had a particularly aggressive form of the disease. This patient was the youngest at 29 years and only lived 18 months after diagnosis. Imaging findings in this patient were somewhat different than the other patients. On CT, the patient’s tumor was more solid appearing (82.9 HU) than that of other patients. It was also FDG avid with an SUV_max_ of 7.4. Two other patients had FDG-PET/CT imaging. Their tumors were either not FDG avid or only mildly so. Their disease burden demonstrated lower Hounsfield unit values and a more protracted clinical course which is typical of EMC. This raises the possibility that tumor density on contrast enhanced CT and FDGPET could provide useful prognostic information. FDG-PET uptake has already been shown to have prognostic significance in other types of conventional chondrosarcoma.^16^ This is not to suggest that FDG-PET/CT imaging should be done in all cases of EMC, but rather that it may be useful in patients where there is concern about tumor aggressiveness and in patients with tumor burden that is hyperdense to muscle on contrast enhanced CT.

Our study has several limitations. First, the sample size was small due to the rarity of the disease, particularly in a single institution. Second, some patients presented to our institution after initial imaging and treatment were performed at an outside hospital. Imaging of the primary tumor was therefore not available for some of these cases. Despite the limitations of this study, our findings raise the hypothesis that tumor density on contrast enhanced CT and FDG-PET uptake may correlate with clinical behavior. This requires further exploration. Furthermore, although it is a rare tumor, EMC should be included in one’s differential for extraskeletal soft tissues masses which display little to no internal calcification, peripheral/septal enhancement, and increased T2 prolongation. Imaging of the chest is also crucial given the frequency of lung metastases in patients with this tumor.

## Figures and Tables

**FIGURE 1A.**
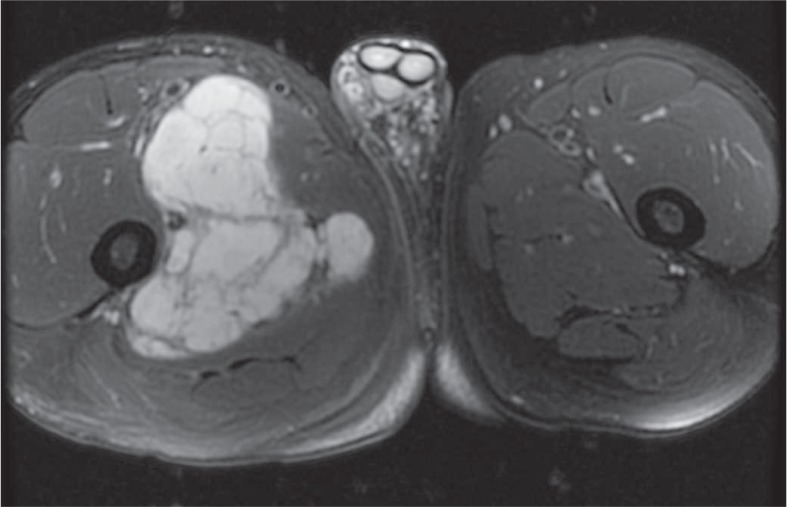
64 year old male with slow growing extraskeletal myxoid chondrosarcoma. Axial T2 fat-saturated (FS) MRI demonstrates a large lobulated T2 hyperintense mass with T2 hypointense internal fibrous septa.

**FIGURE 1B.**
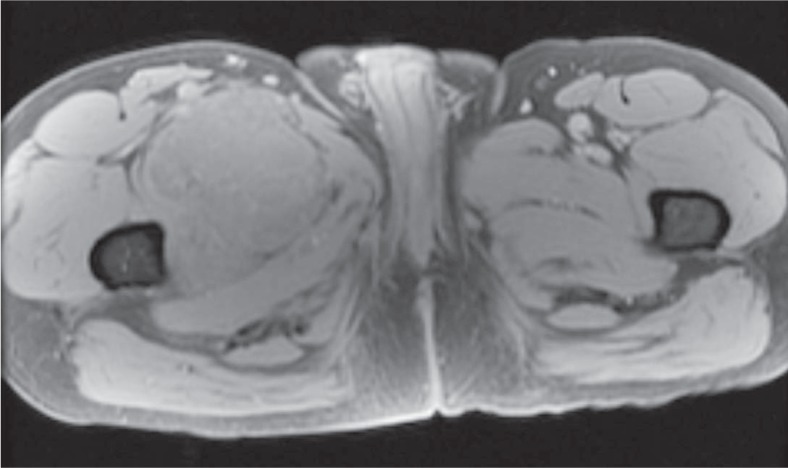
Axial T1 fast spoiled gradient-echo (FSPGR) FS pre-contrast MRI shows that this tumor is isointenese to muscle on T1 weighted images.

**FIGURE 1C.**
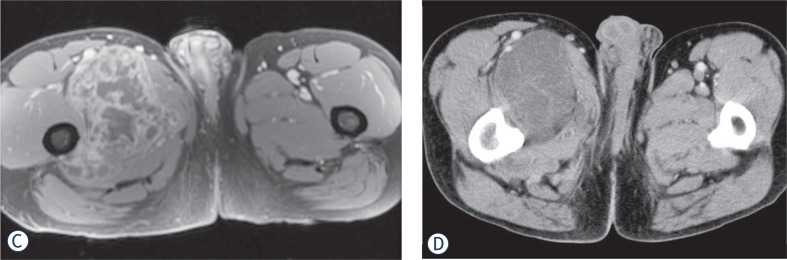
Axial T1 FSPGR FS post-contrast MRI demonstrates peripherhal/septal contrast enhancement. **(D).** Axial contrast enhanced CT shows that this mass is slightly hypodense to muscle.

**FIGURE 1E.**
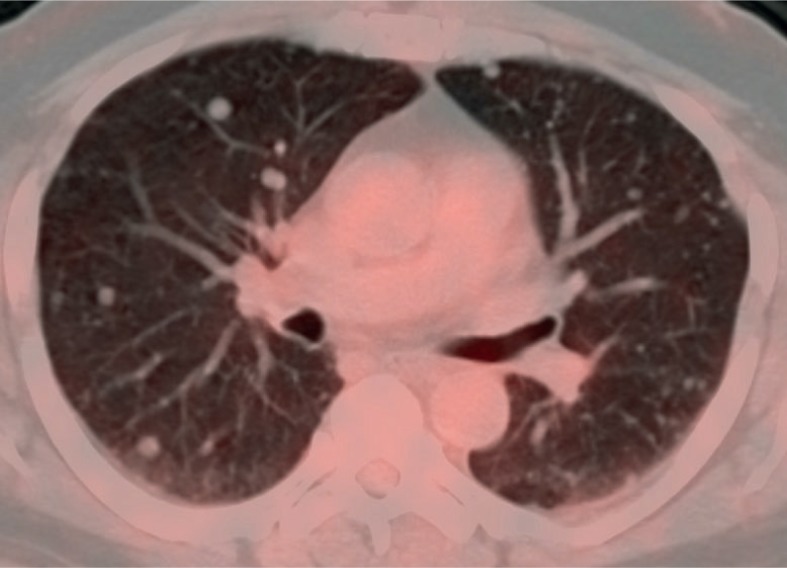
FDG-PET/CT images show no significant FDG uptake in numerous lung metastases.

**FIGURE 3A.**
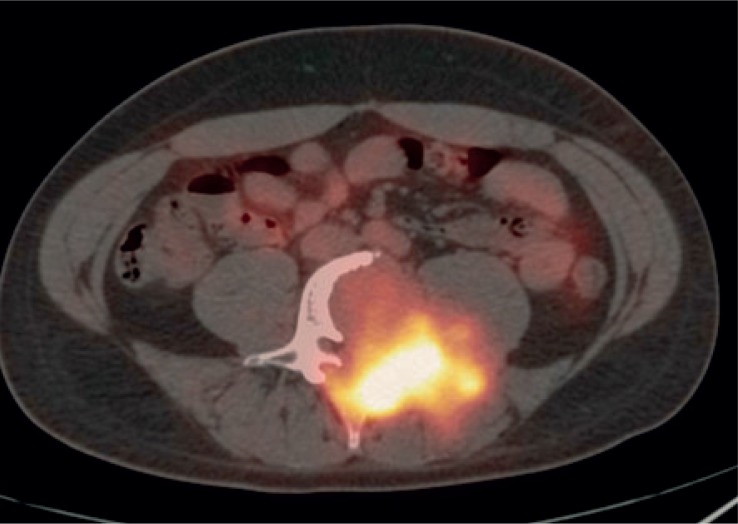
29 year old male with unusually aggressive extraskeletal myxoid chondrosarcoma presenting in the calf. Axial FDG-PET/CT demonstrating significant FDG uptake (SUV_max_ 7.4) in bone metastasis in a patient with high density tumor burden.

**FIGURE 3B.**
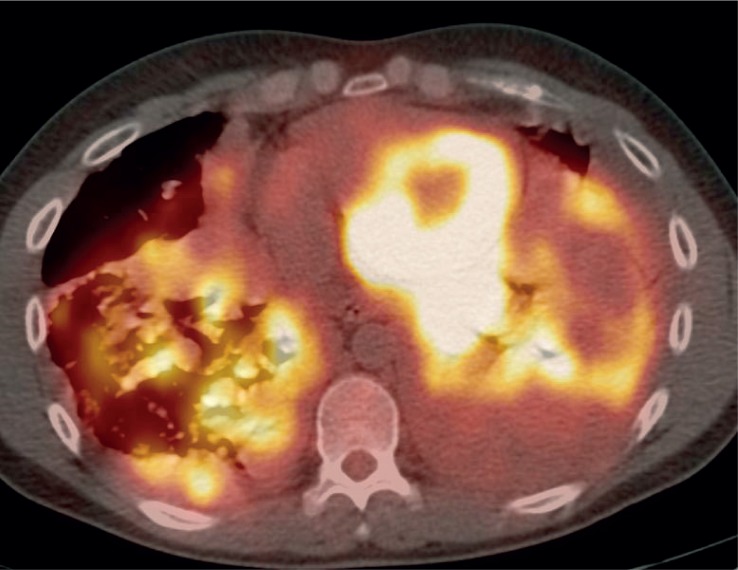
Axial FDG-PET/CT also showing significant FDG uptake in pulmonary and pleural metastases.

**FIGURE 3C.**
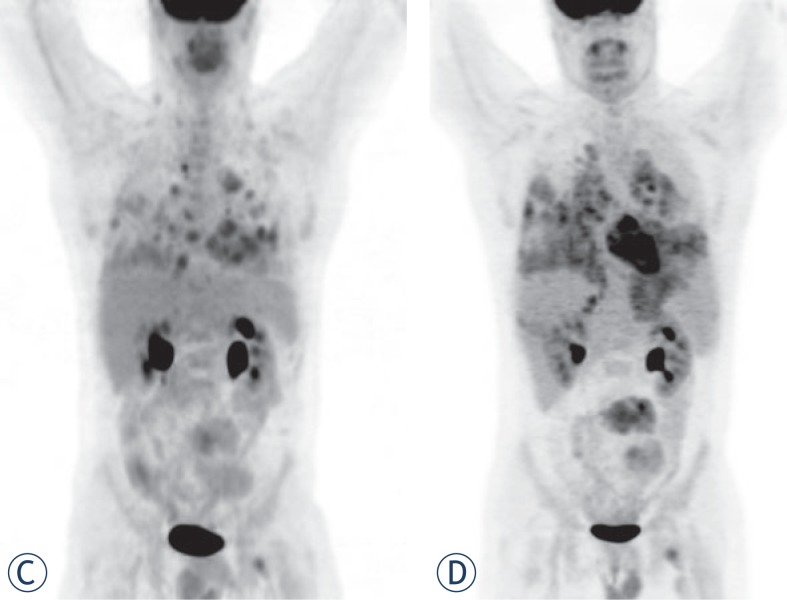
FDG PET study on October 2008 demonstrating tumor burden. **(D).** FDG PET study on December 2008 shows rapid evolution of tumor burden from October 2008.

**FIGURE 4A.**
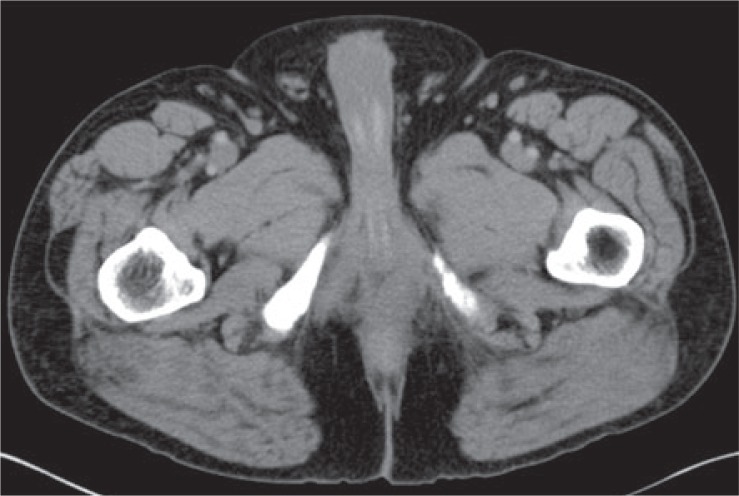
62 year old male with extraskeletal myxoid chondrosarcoma in the pelvis. Axial contrast enhanced CT showing locally recurrent tumor in the left perineum that is isointense to muscle.

**FIGURE 4B.**
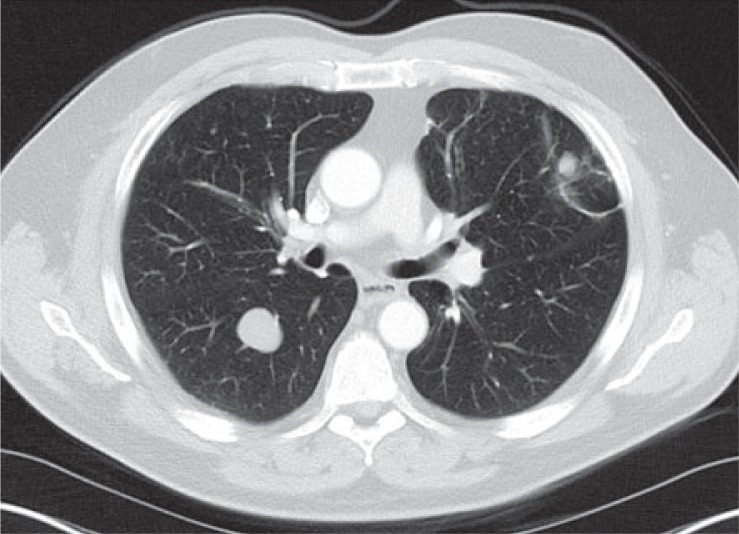
Numerous, large lung metastases.

**FIGURE 4C.**
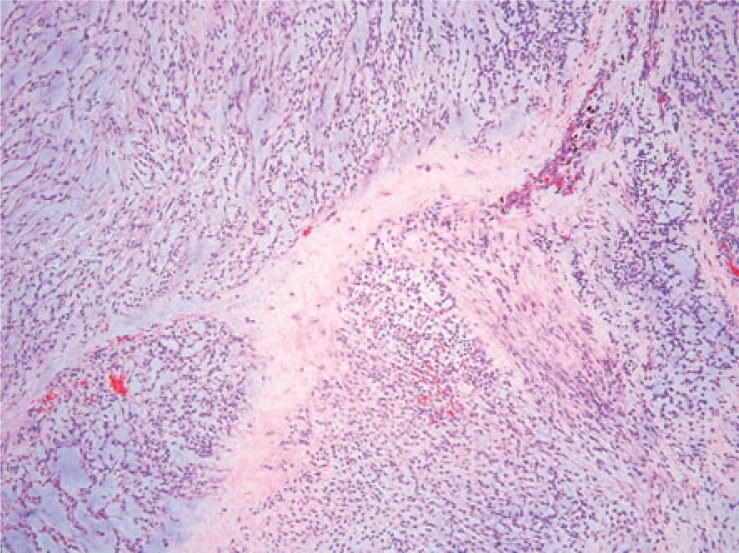
The tumor shows a lobulated appearance.

**FIGURE 4D.**
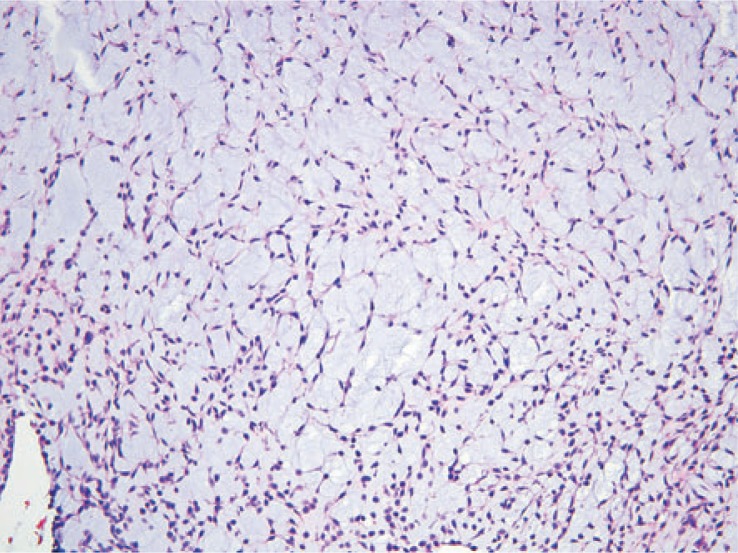
The tumor cells are arranged in a reticular architecture with abundant myxoid stroma.

**FIGURE 4E.**
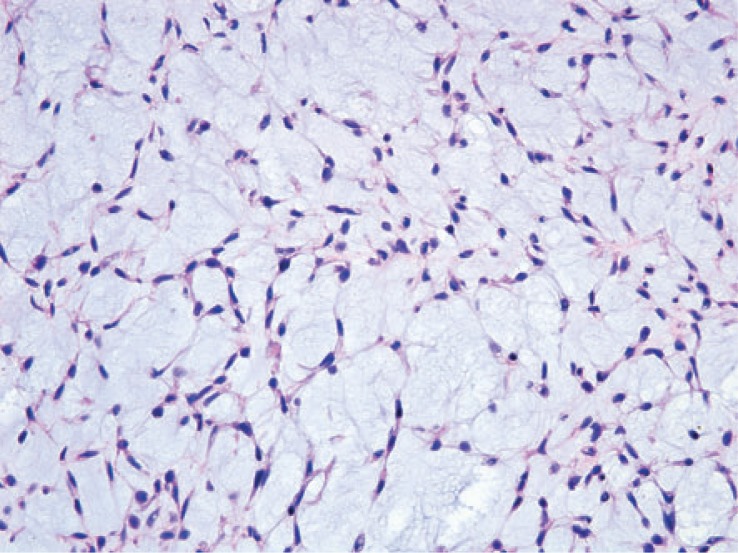
The tumor is composed of bland and uniform spindle cells with hyperchromatic nuclei and delicate eosinophilic cytoplasm.

**FIGURE 4F.**
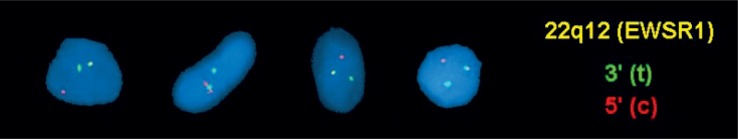
Fluorescence in situ hybridization (FISH) using break-apart probes shows EWSR1 rearrangement at 22q12. Note the separation of the red (centromeric) and green (telomeric) probes

**TABLE 1.** Clinical features of patients with extraskeletal myxoid chondrosarcoma

**Case**	**Age**	**Gender**	**Primary site**	**Tumor size (cm)**	**Time to recurrence (months)**	**Time from diagnosis to metastases (months)**	**Survival after diagnosis (months)**
1	68	M	Thigh	3.3		25	
2	67	M	Foot	4.2		40	
3	53	M	Thigh	7		50	
4	43	M	Thigh	9.5		0	
5	29	M	Calf	12		10	18
6	68	F	Thigh	13		0	
7	64	M	Thigh	14		0	81
8	44	F	Thigh	15		0	
9	73	M	Thigh	18	81	93	143
10	32	M	Spine	4.3			
11	59	F	Spine	6.6		0	
12	54	M	Pelvis	8	4	4	
13	62	M	Pelvis	10	72	50	

F = female; M = male

**TABLE 2.** Location of metastatic disease

**Site**	**Total number of patients**
Lung Pleura/parenchyma	12
Bone	3
Abdominal/pelvic nodes	2
Soft tissues	2
Mediastinal nodes	1
Peritoneum	1
Abdominal/pelvic viscera	0

**TABLE 3.** Hounsfield units of primary, recurrent, and metastatic tumor burden

**Case**	**Primary**	**Recurrence**	**Mediastinal nodes**	**Abdominal nodes**	**Retroperitoneum**	**Lung**	**Soft tissue**	**Bone**
1				29.4		25.7		
2						11.0		
3						20.3		
4						14.0		
5			61.1			82.9		56.4
6						8.2		
7	23.4		21.2		33.6	21.4	26.8	
9		72.4			39.7	34.8		32.4
11	30.2		38.1			20.0	53.2	
12		41.0				37.3		
13		32.2				16.9		

Hounsfield units were measured in the center of the lesion on contrast enhanced CTs. If patients had multiple studies, the study with the largest tumor burden was used. For patients with multiple lung or nodal metastases, the average Hounsfield unit is provided. One patient had lung metastases that were too small to accurately measure.

**TABLE 4.** Density and standard uptake value (SUV)_max_ of patients with PET imaging

	**Survival after diagnosis**	**SUV_max_**	**HU**
Case 7	81	0	33.6
Case 12		2.8	41.0
Case 5	18	7.4	82.9

Highest Hounsfield unit (HU) measured including primary, recurrent, or metastatic tumor burden on contrast enhanced CT.
